# F35. PRESCRIPTION PATTERN OF ANTIPSYCHOTICS FOR CHILDREN AND ADOLESCENTS WITH SCHIZOPHRENIA IN KOREA BASED ON NATIONWIDE DATA

**DOI:** 10.1093/schbul/sby017.566

**Published:** 2018-04-01

**Authors:** Kee Jeong Park, Jung Sun Lee, Hyo-Won Kim

**Affiliations:** Asan Medical Center, University of Ulsan College of Medicine

## Abstract

**Background:**

This study aimed to analyze the extent and pattern of antipsychotic prescription for Korean children and adolescents with schizophrenia using population-based data.

**Methods:**

Our data was retrieved from the Korean National Health Insurance Review & Assessment Service-National Sample for 2013, which was a stratified sampling from the entire population under the Korean national health insurance program. Among 0.2 million children and adolescents aged 6–18 years from data, subjects who had received any antipsychotic medication in the year were investigated for the prescribed medication and concomitant psychotropic medication.

**Results:**

A total of 91 children and adolescents (mean age 16.2 ± 2.2 years, 53 boys) received antipsychotic medication. Among the prescriptions, risperidone (n = 59, 35.3%) and aripiprazole (n = 34, 20.4%) were the two most frequently prescribed antipsychotics. Of 91 patients, 80 (87.9%) have prescribed with antipsychotic monopharmacy (mean 134.9 ± 11.1 day), 33 (36.3%) with bipharmacy (mean 136.9 ± 20.2 days), and 12 (13.2%) with polypharmacy (more than three antipsychotics) (mean 32.5 ± 7.3 days) in the year. Mood stabilizers (n=48, 52.7%), and antidepressants (n=35, 38.5%) were co-medicated in the year.

**Discussion:**

Our study shows the prescription pattern of antipsychotics for children and adolescents with schizophrenia in Korea.

